# HIV self‐testing partially filled the HIV testing gap among men who have sex with men in China during the COVID‐19 pandemic: results from an online survey

**DOI:** 10.1002/jia2.25737

**Published:** 2021-05-26

**Authors:** Hongbo Jiang, Yewei Xie, Yuan Xiong, Yi Zhou, Kaihao Lin, Yao Yan, Joseph Tucker, Jason J Ong, Dan Wu, Fan Yang, Weiming Tang

**Affiliations:** ^1^ Department of Epidemiology and Biostatistics, School of Public Health Guangdong Pharmaceutical University Guangzhou China; ^2^ Dermatology Hospital Southern Medical University Guangzhou China; ^3^ The Institute of Global Health and STD Southern Medical University Guangzhou China; ^4^ University of North Carolina at Chapel Hill Project‐China Guangzhou China; ^5^ Zhuhai Center for Disease Control and Prevention Zhuhai China; ^6^ Department of Clinical Research The London School of Hygiene & Tropical Medicine London United Kingdom; ^7^ Central Clinical School Monash University Melbourne Australia; ^8^ Faculty of Infectious and Tropical Diseases London School of Hygiene & Tropical Medicine London United Kingdom

**Keywords:** men who have sex with men, HIV self‐testing, facility‐based HIV testing, COVID‐19

## Abstract

**Introduction:**

HIV self‐testing (HIVST) is a useful strategy to promote HIV testing among key populations. This study aimed to understand HIV testing behaviours among men who have sex with men (MSM) and specifically how HIVST was used during the coronavirus disease 2019 (COVID‐19) measures in China when access to facility‐based testing was limited.

**Methods:**

An online cross‐sectional study was conducted to recruit men who have sex with men (MSM) in China from May to June of 2020, a period when COVID‐19 measures were easing. Data on socio‐demographic characteristics, sexual behaviours and HIV testing in the three months before and during COVID‐19 measures (23 January 2020) were collected. Chi‐square test and logistic regression were used for analyses.

**Results:**

Overall, 685 MSM were recruited from 135 cities in 30 provinces of China, whose mean age was 28.8 (SD: 6.9) years old. The majority of participants self‐identified as gay (81.9%) and had disclosed their sexual orientation (66.7%). In the last three months, 69.6% ever had sex with men, nearly half of whom had multiple sexual partners (47.2%). Although the overall HIV testing rates before and during COVID‐19 measures were comparable, more MSM self‐tested for HIV during COVID‐19 measures (52.1%) compared to before COVID‐19 measures (41.6%, *p* = 0.038). Fewer MSM used facility‐based HIV testing during COVID‐19 measures (42.9%) compared to before COVID‐19 measures (54.1%, *p* = 0.038). Among 138 facility‐based testers before COVID‐19 measures, 59.4% stopped facility‐based testing during COVID‐19 measures. Among 136 self‐testers during COVID‐19 measures, 58.1% had no HIV self‐testing before COVID‐19 measures. Multivariable logistic regression showed that having sex with other men in the last three months (adjusted odds ratio, a*OR* = 2.04, 95% CI: 1.38 to 3.03), self‐identifying as gay (a*OR* = 2.03, 95% CI: 1.31 to 3.13), ever disclosing their sexual orientation (a*OR* = 1.72, 95% CI: 1.19 to 2.50) and tested for HIV in three months before COVID‐19 measures (a*OR* = 4.74, 95% CI: 3.35 to 6.70) were associated with HIV testing during COVID‐19 measures.

**Conclusions:**

Facility‐based HIV testing decreased and HIVST increased among MSM during COVID‐19 measures in China. MSM successfully accessed HIVST as substitute for facility‐based testing, with no overall decrease in HIV testing rates.

## 1 Introduction

In response to coronavirus disease 2019 (COVID‐19), China issued comprehensive COVID‐19 measures on 23 January, 2020. Shortly thereafter, the highest response level to the public health emergency was initiated by all provinces in mainland China [1, 2]. A series of COVID‐19 measures were applied including city lockdown, travel restriction, social distancing, quarantine and physical isolation [1, 2]. Although the non‐pharmaceutical measures were effective to contain COVID‐19 [3], they also resulted in unintended barriers to access routine medical services, including HIV testing [4].

Despite global efforts to control HIV among key populations, HIV disproportionately affects men who have sex with men (MSM) around the world [5]. In China, HIV prevalence among MSM has reached 8.0% in 2015 [6]. However, barriers such as perceived lack of confidentiality, stigma and discrimination result in low access to routine HIV testing services among MSM [7, 8, 9]. For example only 62.3% of MSM from an online survey in 2016 had ever tested for HIV in China [10]. During the COVID‐19 pandemic, challenges in accessing routine HIV testing in facility‐based services were added to existing barriers which could further expand the testing gap [11]. Low HIV testing rates could result in worse clinical outcomes and increased onward transmission for those people living with HIV who are undiagnosed [11].

HIV self‐testing (HIVST) has the potential to increase HIV testing uptake among key populations, and is recommended by the World Health Organization (WHO) as an approach to routine HIV testing services [12]. Owing to its confidentiality and convenience, HIVST has the potential to be used to maintain HIV testing rates during the pandemic [4, 11, 13, 14]. However, the role that HIVST played during the pandemic has not been well described in China.

The aims of this cross‐sectional online survey were to understand HIV testing behaviours among MSM, and how HIVST was used during the COVID‐19 pandemic among MSM in China.

## 2 Methods

### 2.1 Study design and participants

This online cross‐sectional study was conducted among Chinese MSM from 18 May to 2 June, 2020, a period when COVID‐19 measures in China were easing. Twenty‐third January 2020 was used as a cut‐off date of two periods, namely before and during COVID‐19 measures, as China started to issue comprehensive COVID‐19 measures since that day. Banner ads to the online survey were placed in a major social networking platform (WeChat, like twitter, one of the largest social media platforms in China with an estimate of one billion monthly active users [15]) to reach MSM across the nation. The ads which were geo‐targeted within China briefly introduced the aims of the survey and clarified the potential participants (i.e. MSM). If participants completed the questionnaire and participated in a lottery draw, they would have the chance to get a T‐shirt provided by a community‐based organization (CBO). Potential participants clicked the link of the survey and signed an electronic written inform consent and were screened for eligibility. Inclusion criteria were individuals who were born biologically as a male, self‐identified as a male, aged 18 or over and ever engaged in sex with a man, while each participant can only attend the survey once restricted by phone number and IP address. IP address was also used to identify individuals residing in China. With an average HIV testing rate in the last three months among MSM (*p*) of 20% [16], a precision error (d) of 0.15*P* and a confidence level of 95%, the required sample size was expected to be 683. This study was approved by the Ethics Committee of Zhuhai Center for Diseases Control and Prevention (CDC).

### 2.2 Measures

Data on sociodemographic and behavioural characteristics were collected. Behavioural variables in the last three months before the survey included ever having sex with men, having more than one sexual partner (multiple sexual partners), condom use during the last sexual intercourse with a male partner, recreational drug use and use of social network apps (Blued, etc.) to seek sexual partners. Participants were asked to report whether they had fewer sexual partners, used condom less frequently and sought sexual partners using apps more frequently during COVID‐19 measures compared to before COVID‐19 measures. Data on HIV testing history three months before and during COVID‐19 measures were collected. Items on participants’ attitudes towards HIVST during COVID‐19 measures included whether HIVST kits were easy to get and use and whether HIVST met their testing needs.

### 2.3 Statistical analysis

Descriptive analysis was conducted to present the sociodemographic and behavioural characteristics and HIV testing related variables. Chi‐squared test was applied to compare the HIV testing before and during COVID‐19 measures. Multivariable logistic regression was conducted and reported as an adjusted *odds ratio* (*aOR*) and 95% *confidence interval* (CI) after adjusting for variables statistically significant in univariate analyses. Hosmer–Lemeshow test was used to assess the goodness‐of‐fit of the model. Data analyses were performed using SPSS software (version 20.0).

## 3 RESULTS AND DISCUSSION

In total, 804 individuals completed the survey though 119 were excluded (20 were aged below 18, 19 were born biologically as female, 42 did not self‐identify as male, four had mobile IP addresses outside China and 34 had not had sex with a man). After applying exclusion criteria, 685 MSM from 135 cities in 30 provinces of China were included in this analysis.

The mean age of the participants was 28.8 (SD 6.9) years old. The majority of the participants self‐identified as gay (81.9%), never married (87.3%), attended a college and above (79.1%) and not a student (85.3%). More than half (52.1%) earned an annual income of less than $USD 5000. The proportion of participants who disclosed their sexual orientation to others, ever had HIV testing three months before COVID‐19 measures, and ever had sex with other men in the last three months before the survey were 66.7%, 37.2% and 69.6% respectively. Among 477 participants who ever had sex with other men in the last three months before the survey, 225 (47.2%) had multiple sexual partners and 292 (61.2%) had fewer sexual partners compared to before COVID‐19 measures. The proportion of people who used a condom during their last sexual intercourse with a male partner in the last three months before the survey was 71.3%, and 24.1% reported less frequent condom use compared to before COVID‐19 measures (Table 1).

**Table 1:** Characteristics of men who have sex with men included in the online survey in China, 2020 (N = 685)

Characteristics	n (%)	Characteristics	n (%)
Sex		Ever had sex with men in the last three months	
Male	685 (100.0)	Yes	477 (69.6)
Age, years		No	208 (30.4)
≤27	358 (52.3)	Multiple sexual partners in the last three months (n = 477)	
>27	327 (47.7)	Yes	225 (47.2)
Marital status		No	252 (52.8)
Engaged/married	49 (7.2)	Less number of sexual partners compared to before COVID‐19^a^ measures (n = 477)	
Never married	598 (87.3)	Yes	292 (61.2)
Divorced/Separated/Widowed	38 (5.5)	No	185 (38.8)
Highest level of education		Sex role	
High school or below	143 (20.9)	Insertive	194 (40.7)
Some college	173 (25.3)	Receptive	163 (34.2)
University	305 (44.5)	Both	120 (25.2)
Postgraduate	64 (9.3)	Used a condom during last sexual intercourse with a male partner (n = 477)	
Annual income levels, USD		Yes	340 (71.3)
≤5000	357 (52.1)	No	118 (24.7)
>5000	328 (47.9)	Unsure	19 (4.0)
Student		Less frequent condom use compared to before COVID‐19 measures (n = 477)	
Yes	101 (14.7)	Yes	115 (24.1)
No	584 (85.3)	No	362 (75.9)
Sexual Orientation		Recreational drug use in the last three months (n = 477)	
Gay	561 (81.9)	Yes	157 (32.9)
Heterosexual/Bisexual/Unsure	124 (18.1)	No	320 (67.1)
Sexual orientation disclosure to others		Seeking sexual partners using apps^b^ in the last three months (n = 477)	
Yes	457 (66.7)	Yes	370 (77.6)
No	228 (33.3)	No	107 (22.4)
Ever tested for HIV three months before COVID‐19^a^ measures		More frequent to seek sexual partners using apps compared to before COVID‐19 measures (n = 370)	
Yes	255 (37.2)	Yes	106 (28.6)
No	430 (62.8)	No	264 (71.4)

^a^ COVID‐19 refers to coronavirus disease 2019

^b^ apps refers to Blued, Aloha, Jack'd, WeChat, etc.

**Figure 1:**
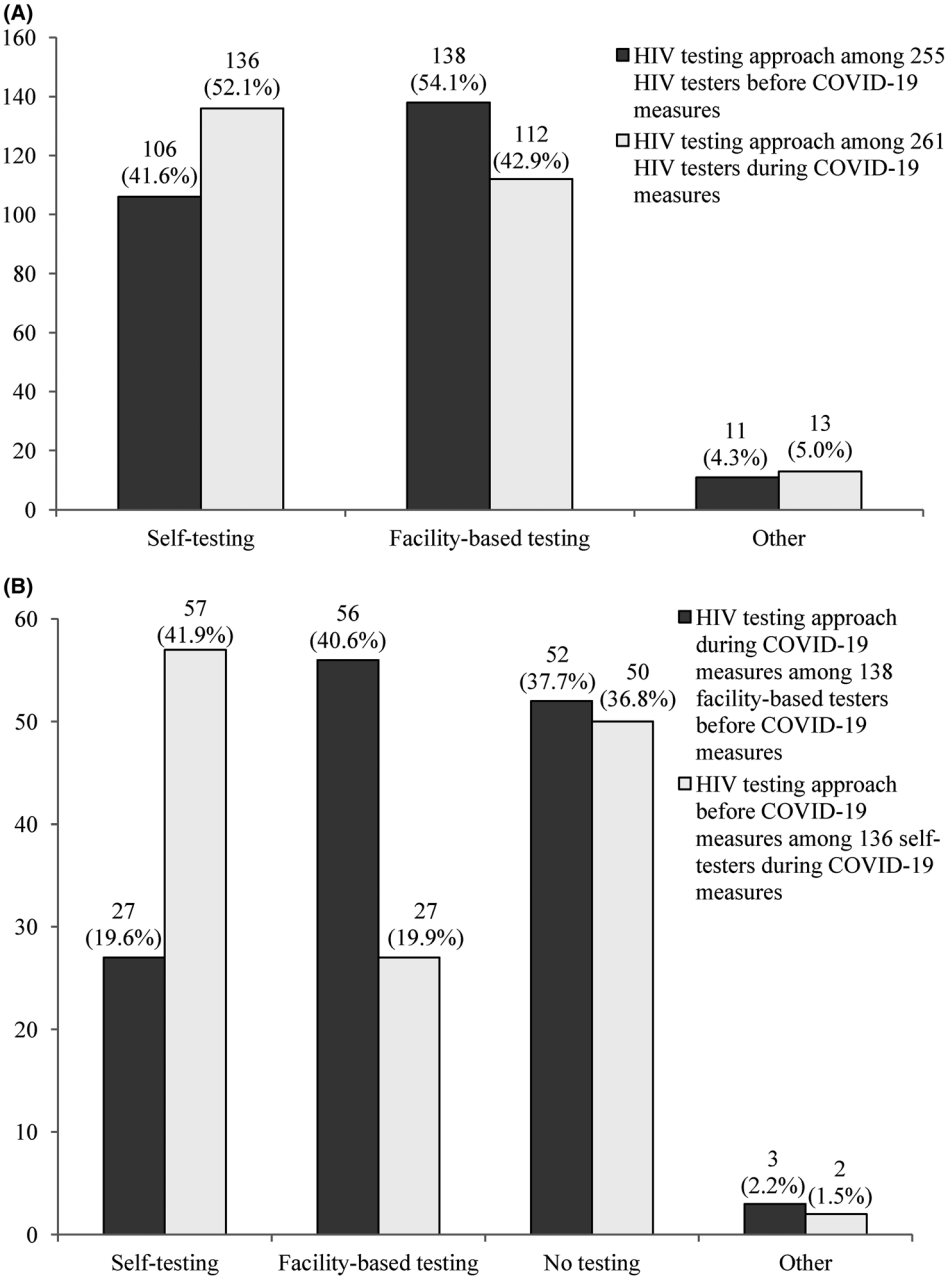
Changes in HIV testing approach before and during COVID‐19† measures. **(A)** HIV testing approach before and during COVID‐19 measures. **(B)** Changes in facility‐based HIV testing and HIV self‐testing before and during COVID‐19^†^ measures. ^†^COVID‐19 refers to coronavirus disease 2019.

Overall, 261 (38.1%) participants tested for HIV during COVID‐19 measures. HIV testing within three months before and during COVID‐19 measures was comparable (255 vs. 261), whereas a higher proportion of participants self‐tested for HIV during COVID‐19 measures (52.1% vs. 41.6%, *χ^2^* = 6.521, *p* = 0.038), and lower proportion of participants underwent facility‐based testing during COVID‐19 measures (42.9% vs. 54.1%, *χ^2^* = 6.521, *p* = 0.038, Figure 1A). Among 138 participants who underwent facility‐based testing three months before COVID‐19 measures, 82 (59.4%) stopped facility‐based testing during COVID‐19 measures. Among 136 participants who self‐tested for HIV during COVID‐19 measures, 79 (58.1%) had no HIVST three months before COVID‐19 measures (Figure 1B).

The multivariable logistic regression revealed that individuals who had sex with other men (a*OR* = 2.04, 95% CI: 1.38 to 3.03) in the last three months, self‐identified as gay (a*OR* = 2.03, 95% CI: 1.31 to 3.13), ever disclosed their sexual orientation (a*OR* = 1.72, 95% CI: 1.19 to 2.50) and ever tested for HIV (a*OR* = 4.74, 95% CI: 3.35 to 6.70) three months before COVID‐19 measures were more likely to undergo HIV testing during COVID‐19 measures (Table 2).

**Table 2:** Factors associated with HIV testing during COVID‐19^a^ measures among men who have sex with men in China

Characteristics	n (%)	HIV testing					
		n (%)	χ^2^	*p*	aOR^b^	95% CI	*p*
Sexual Orientation			4.828	0.028			0.001
Gay	561 (81.9)	203 (36.2)			2.03	1.31 to 3.13	
Heterosexual/Bisexual/Unsure	124 (18.1)	58 (46.8)			1.00		
Ever had sex with men in the last three months			23.367	<0.001			<0.001
Yes	477 (69.6)	210 (44.0)			2.04	1.38 to 3.03	
No	208 (30.4)	51 (24.5)			1.00		
Sexual orientation disclosure to others			13.336	<0.001			0.004
Yes	457 (66.7)	196 (42.9)			1.72	1.19 to 2.50	
No	228 (33.3)	65 (28.5)			1.00		
HIV testing three months before COVID‐19 measures			94.849	<0.001			<0.001
Yes	255 (37.2)	157 (61.6)			4.74	3.35 to 6.70	
No	430 (62.8)	104 (24.2)			1.00		
Multiple sexual partners in the last three months (n = 477)			6.637	0.010			0.105
Yes	225 (47.2)	113 (50.2)			1.39^c^	0.93 to 2.07	
No	252 (52.8)	97 (38.5)			1.00		

CI, confidence interval.

^a^ COVID‐19 refers to coronavirus disease 2019

^b^ aOR: adjusted odds ratio, odds ratio adjusting for sexual orientation, ever had sex with men in the last three months, sexual orientation disclosure to others, HIV testing three months before COVID‐19 measures

^c^ odds ratio adjusting for sexual orientation, sexual orientation disclosure to others, HIV testing three month before COVID‐19 measures.

During COVID‐19 measures, the reasons for not testing included: limited access to routine facility‐based testing services (42.3%), being worried about getting COVID‐19 when going out for test (40.1%), limited access to self‐testing kits, (12.0%) and other (5.6%). The majority of the 136 participants who used HIVST during the COVID‐19 measures reported that self‐testing kits were easy to get (87.5%), was an effective alternative to facility‐based testing (93.4%), was easy to use (97.8%) and met their testing needs (91.2%).

In response to the COVID‐19 pandemic, many countries have implemented COVID‐19 measures, which also impacted the access to routine healthcare services. Our study found that limited access to facility‐based HIV testing services and fear of getting COVID‐19 led to a decrease in facility‐based testing during COVID‐19 measures among MSM using social network apps in China.

Facility‐based HIV testing decreased during COVID‐19 measures among MSM in our study. In addition, more than half of the facility‐based testers before COVID‐19 measures stopped facility‐based testing during COVID‐19 measures. Limited access to routine HIV testing services and fear of getting COVID‐19 were the main reasons for not testing during COVID‐19 measures. Since the outbreak of COVID‐19, healthcare systems have been overwhelmed by patients infected with COVID‐19, and thus access to routine healthcare services decreased [17, 18]. COVID‐19 measures impeded access to HIV testing services and COVID‐related stigma created barriers to visit facility‐based HIV testing services [14, 19]. Disruption to HIV programmes caused by COVID‐19 may have a substantial effect on HIV incidence and population‐level mortality [20]. Therefore, it is crucial to maintain HIV testing during the COVID‐19 pandemic.

HIVST increased during COVID‐19 measures among MSM in our study. Although COVID‐19 measures were implemented, we did not observe a reduction in overall HIV testing among Chinese MSM. One important reason for this was that HIV self‐testing was used as an alternative approach, becoming the dominant means of HIV testing during COVID‐19 measures. The decentralized nature of HIVST combined with the use of digital health services make HIV self‐testing more accessible [21], especially when facility‐based HIV testing services are limited. The uptake of HIVST could reduce the visits to health facilities when social distancing measures were implemented and also prevent overwhelming existing health facilities. In addition, the attitudes to HIVST during COVID‐19 measures suggested the feasibility of HIVST to fill the testing gap during the pandemic.

This study has several implications for maintaining HIV services during the pandemic. First, HIV testing demand was sustained during the pandemic even when access to facility‐based testing was limited. Policy makers should ensure that testing services continue in alternate forms. Second, HIVST could be a feasible and effective way to fill the testing gap during the pandemic. Strategies to promote HIVST including but not limited to improving access and expanding coverage, should be tailored to meet the testing needs of MSM. Third, although HIVST filled the HIV testing gap during the pandemic, linkage to care remained a challenge for self‐testers [22]. Targeted measures should be implemented to strengthen the linkage between HIVST and antiretroviral treatment initiation facilities, when the situation could be worse during the pandemic [4].

This study had some limitations. First, no causal associations could be inferred for the cross‐sectional design of this study. Second, we should pay attention to the possibility of selection bias, as our only recruited participants tend to be young and well‐educated MSM. Social network apps‐using MSM were more likely to be engaged in risky sexual behaviours, and to have tested for HIV in lifetime compared to non‐apps‐using MSM [23]. In order to expand and diversify this sample, we sent the link of the ads to local CDCs and CBOs to help promote the recruitment. Nevertheless, access to HIV testing, getting an HIV test and trouble getting an HIV test did not differ between the older and the younger subgroups [14]. However, more attention should be paid to MSM with less education and without access to technologies (i.e. smart phones and computers) in contexts with high social and economic inequality, who may not have the same access to HIV testing and HIVST as the participants in the current study. Third, the effect of COVID‐19 measures on routine HIV testing services could be underestimated since our survey was conducted when COVID‐19 measures were easing. Four, there could be recall bias to collect data three months before COVID‐19 measures. Nevertheless, the cut‐off date was very close to Spring Festival, which could help participants’ memory recall.

## 4 Conclusions

During the COVID‐19 pandemic, limited access to HIV testing services and fear of getting COVID‐19 led to a decrease in facility‐based testing among study participants while the lockdown measures were in place. However, more HIV self‐testing was observed. Our study highlights that HIVST can be effectively used to meet the ongoing demands for HIV testing among MSM during the COVID‐19 pandemic.

## Competing interests

The authors have declared no conflict of interest.

## Authors’ contributions

HJ and WT designed the research study. HJ, YXie, YXiong and YZ contributed to acquisition of data. HJ, KL and YY analysed and interpreted the data. HJ drafted the manuscript. WT, JT, JJO, DW and FY revised the manuscript critically for important intellectual content. All the authors reviewed and approved the manuscript.
